# A Sub‐Diffraction‐Limit Dimension All‐Plasmonic Optical Memory Using Non‐Linear Photochromism

**DOI:** 10.1002/advs.202502890

**Published:** 2025-05-08

**Authors:** Shuichi Toyouchi, Mathias Wolf, Kenji Hirai, Yasuhiko Fujita, Tomoko Inose, Beatrice Fortuni, Eduard Fron, Johan Hofkens, Steven De Feyter, James Hutchison, Tsuyoshi Fukaminato, Hiroshi Uji‐i

**Affiliations:** ^1^ Department of Chemistry KU Leuven, Celestijnenlaan 200F Leuven 3001 Belgium; ^2^ Research Institute for Electronic Science (RIES) Hokkaido University N20W10, Kita ward Sapporo Hokkaido 001–0020 Japan; ^3^ Toray Research Center Inc., Sonoyama 3‐3‐7 Otsu Shiga 520–8567 Japan; ^4^ Institute for Integrated Cell‐Material Science (WPI–iCeMS) Kyoto University Yoshida Sakyo–ku Kyoto 606–8501 Japan; ^5^ Max Plank Institute for Polymer Research D‐55128 Mainz Germany; ^6^ School of Chemistry and ARC Centre of Excellence in Exciton Science University of Melbourne Parkville Victoria 3010 Australia; ^7^ Graduate School of Science and Technology Kumamoto University 2‐39‐1 Kurokami Kumamoto 860–8555 Japan

**Keywords:** chemically synthesized silver nanowire, nonlinear plasmonics, optical memory, photochromism, plasmonic waveguide

## Abstract

The development of compact, high‐speed, and energy‐efficient optical memories remains a significant challenge in photonic and plasmonic technologies. Conventional optical memories are inherently limited by light diffraction, restricting miniaturization and causing inefficient energy transfer. A promising strategy to overcome these limitations is using propagating surface plasmon polaritons (SPPs), enabling the confinement and propagation of optical fields along metal interfaces, and allowing photonic devices to scale down to sub‐diffraction‐limit dimensions. This work presents an all‐plasmonic optical memory system based on silver nanowires (AgNWs) coated with photochromic diarylethene (DAE). By utilizing SPPs, reversible Write/Erase functions are achieved through multiphoton excitation, modulating the photostationary state of DAE. The refractive index changes regulate SPP propagation efficiency along the AgNW, with the memory state being read via plasmonic second‐harmonic generation. The synergy between nonlinear plasmonics in AgNWs and the photochromic properties of DAE enables complete memory operations, including writing, erasing, and reading ON/OFF states. This sub‐diffraction‐limit system paves the way for ultra‐compact, molecular‐scale optical memory devices.

## Graphical abstract

An all‐plasmonic optical memory is demonstrated by using nonlinear plasmonics on a sub‐diffraction limit plasmonic nanowire waveguide coated with a photochromic diarylethene that supports the complete memory processes, “Write”, “Erase”, and “Readout” the memory ON/OFF states.

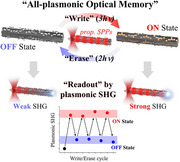

## 1 Introduction

Optical memory can serve as a data storage medium, utilizing photons as carriers instead of the electrons employed in traditional electronic memory. The use of optical memory enables faster read/write speeds, parallel data processing, and long‐term stability,^[^ ^1^, ^2^ ^]^ making it a promising candidate for next‐generation devices. However, the miniaturization of optical memory is inherently limited by light's diffraction limit. To overcome this, a promising approach is using propagating surface plasmon polaritons (SPPs), emerging from the strong coupling of light with collective electronic oscillations. SPPs enable the confinement and propagation of optical fields at sub‐diffraction‐limit scales along metal interfaces,^[^ ^3^, ^4^ ^]^ facilitating the downscaling of photonic devices to sub‐wavelength dimensions.

In particular, metallic nanowires serve as efficient plasmonic waveguides, confining light to diameters as small as one‐tenth of their free‐space wavelength.^[^ ^5^, ^6^, ^7^, ^8^, ^9^ ^]^ Employing this advantage, plasmonic waveguides can be integrated into memory devices, where SPP propagation is modulated by manipulating the dielectric environment through electro‐optic effects^[^ ^10^, ^11^, ^12^, ^13^ ^]^ or incorporating photo‐ or thermo‐responsive materials.^[^ ^14^, ^15^, ^16^, ^17^ ^]^ However, such designs often rely on additional architectures—external circuits or illumination to adjust resistance levels, complicating the system and limiting integration density. In this context, the development of an all‐plasmonic optical memory capable of performing Write, Erase, and Readout operations through propagating SPPs is highly desirable.

**Figure 1:**
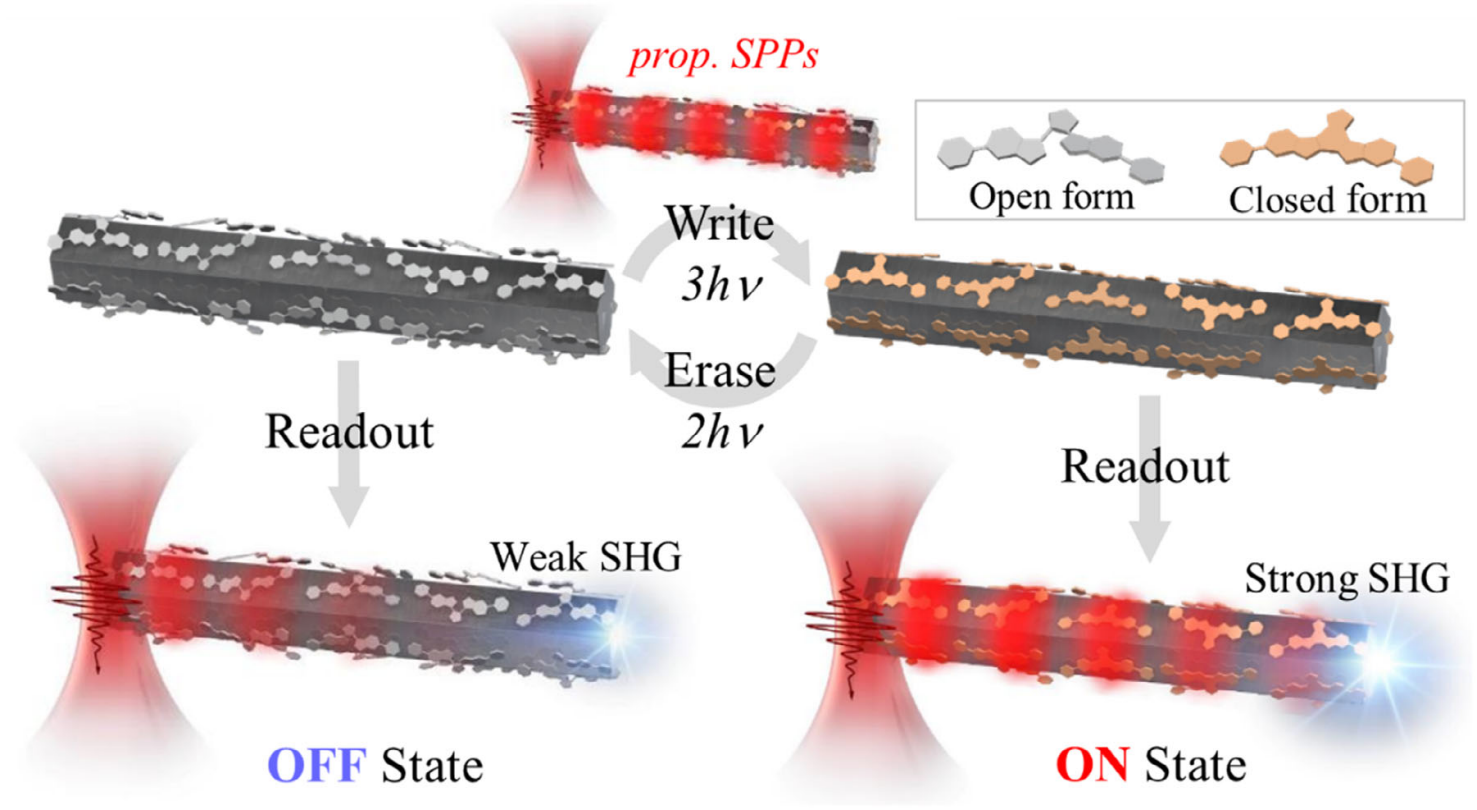
An optical memory system based on AgNW with DAE: Write, Erase, and Readout functions are all achieved by intensity modulation of propagating SPPs launched by laser excitation at the end of a photochrome‐coated AgNW. Write/Erase are achieved by modulating relatively high‐intensity SPPs, inducing multiphoton photochromic reactions, 3‐photon cyclization, and 2‐photon cycloreversion respectively. This sets the refractive index surrounding the nanowire and thus the SPP propagation efficiency (resistance level). To readout this level, the propagation efficiency of relatively low‐intensity SPPs is estimated by SHG at the opposite end of the wire. The Readout SPP does not alter the photochrome equilibrium, which is thermally stable, and thus memory‐retaining in the absence of further multiphoton Write/Erase processes.

Here, we present such a system (**Figure** **1**), employing intensity modulation of near‐infrared (NIR) SPPs propagating on silver nanowires (AgNWs) coated with diarylethene (DAE), a thermally stable yet optically reversible photochromic molecule.^[^ ^18^ ^]^ High‐intensity pulsed laser excitation at one end of the AgNW launches SPPs, inducing multiphoton reactions of DAE along the nanowire surface. The open and closed forms of DAE exhibit distinct power dependencies, allowing modulation of SPP intensity to control the photostationary equilibrium of DAE. Switching the states of DAE alters the refractive index around the AgNW, thereby regulating SPP propagation efficiency analogously to resistance in optical memory (Write/Erase). Additionally, lower‐intensity SPPs can remotely generate plasmonic second‐harmonic generation (SHG) at the distal end,^[^ ^19^ ^]^ enabling straightforward resistance Readout. This deep sub‐diffraction‐limit, optical memory offers a significant advance toward compact, integrated systems for future molecular devices.

## 2 Results and Discussion

### 2.1 Characterizing Multiphotonic Plasmonic Excitation of Chromophores on AgNWs

**Figure 2:**
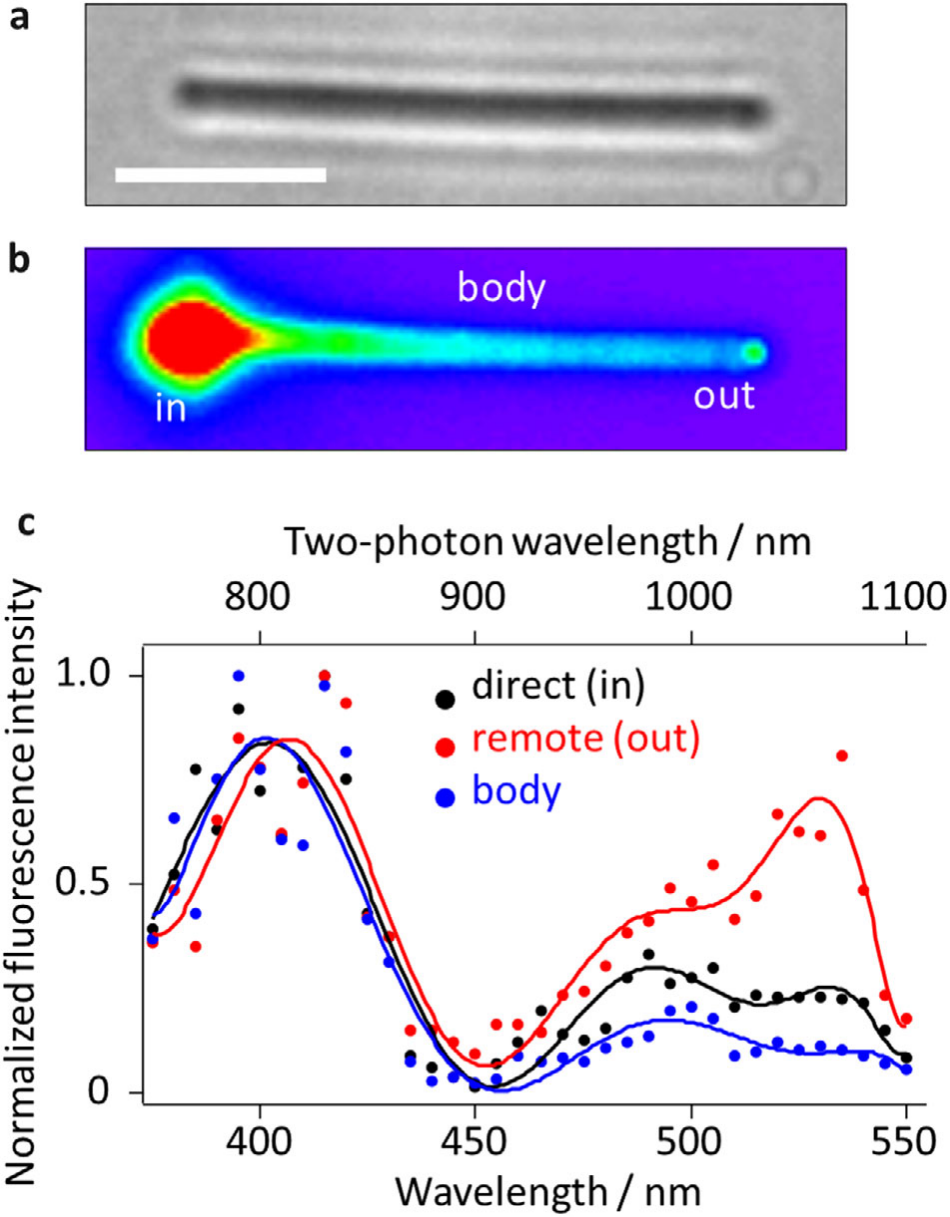
Plasmonic multiphoton excitation of fluorophores on AgNWs. a,b) Optical transmission and fluorescence images, respectively of a AgNW embedded in Rh6G/PVA matrix, under laser excitation (820 nm, 120 fs, 4.36 GW/cm^2^) focused at the left end. The scale bar is 2.5 µm. c) Spatially‐resolved two‐photon excitation spectra of Rh6G measured at the left end (black, direct (in)), the right end (red, remote (out)), and the body (blue) of the AgNW (solid lines are eye guides).

To study SPP‐induced nonlinear optical (NLO) interactions on AgNW, we used rhodamine 6G (Rh6G) as a non‐photochromic fluorophore. AgNWs (average diameter 150 nm, typical length ≈10 µm, **Figure** **2**a) were embedded in a polyvinyl alcohol (PVA) matrix doped with Rh6G. A pulsed NIR laser (820 nm, 120 fs, 4.36 GW/cm^2^) was focused on the left end of AgNW, with polarization aligned to its longitudinal axis (defined as p‐polarized, Figure S1, Supporting Information) to excite propagating SPPs. Rh6G fluorescence was observed along the AgNW with decay (≈12.1 µm, Figure S2, Supporting Information) and at its distal end (Figure 2b). Since Rh6G has negligible absorption at 820 nm, multiphoton excitation of Rh6G is presumed to be mediated by SPPs. The evanescent SPP fields confine the interactions with Rh6G to within tens of nanometers of the surface, with multiphoton events being even more highly surface‐localized.

Fluorescence measured at the left end (“in”), distal end (“out”), and along the body confirms two‐photon excitation of Rh6G, showing a quadratic power dependence (Figure S3, Supporting Information). Note that “excitation” is always at the left end, while, for the fluorescence measurements at the distal end and the AgNW body, “detection” is deliberately separated from the “excitation” in a system referred to as “remote excitation” (Figure S4, Supporting Information). Using wavelengths from 750 to 1100 nm, we obtained Rh6G two‐photon excitation spectra (Figure 2c) dominated by S_0_→S_2_ (400 nm) and S_0_→S_1_ (540 nm) transitions, with the S_0_→S_2_ transition being more prominent than in one‐photon excitation spectrum^[^ ^20^, ^21^ ^]^ (see Section S5, Supporting Information) due to selection rules. The excitation spectra strongly depend on the “detection” position on the AgNW, with the spectrum along the body similar to Rh6G without any AgNW (Figure S5, Supporting Information). However, the two‐photon excitation spectrum at the distal end (“out”) showed significantly higher S_0_→S_1_ transition probability, revealing that Rh6G molecules at the distal end experienced additional one‐photon excitation. This “quasi two‐photon” absorption was attributed to the plasmonic SHG at the AgNW ends,^[^ ^19^ ^]^ producing 540 nm light absorbed in a one‐photon process by Rh6G. At the body of AgNW, SHG was weaker due to the absence of symmetry breaking, and at the excitation end, far‐field effects overshadowed “quasi two‐photon” absorption. These results indicate that AgNW can interact with SPPs in a multi‐photon manner at a sub‐diffraction‐limit dimension, enabling the switching of photochromic molecular state (“Write” and “Erase”). Furthermore, the end‐specific plasmonic SHG offers a means of “Readout” the propagating SPPs along AgNW.

### 2.2 NLO Plasmonic Control of Diarylethene Photochromism on AgNWs

**Figure 3:**
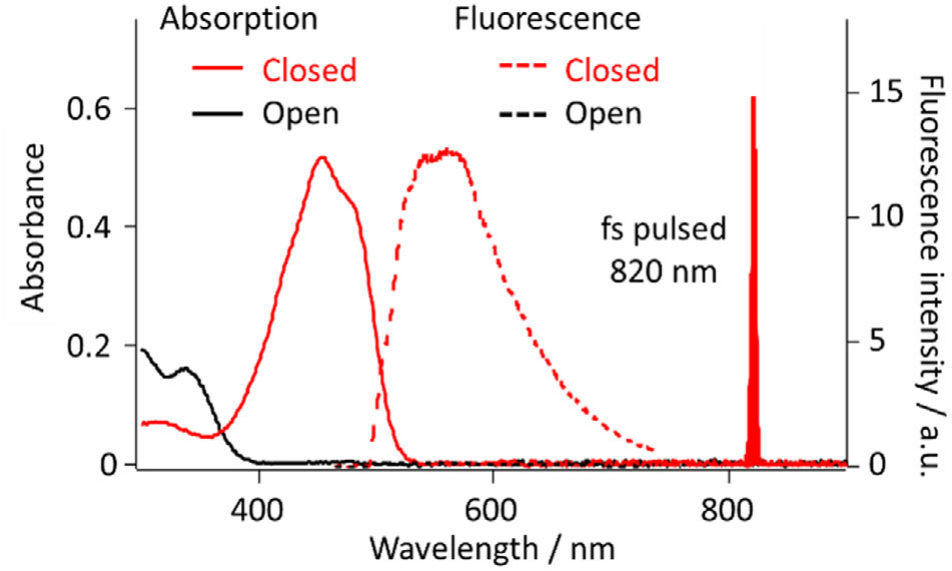
Absorption in isopropanol solution (solid lines) and fluorescence in the PVA matrix (dashed lines) of the DAE, red and black lines represent the closed and open forms, respectively.

**Figure 4:**
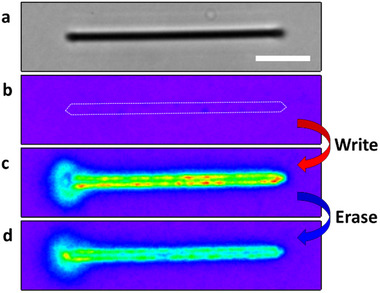
Plasmonic multiphoton control of DAE photochromism on AgNW. a) An optical transmission image of a AgNW. The scale bar is 2.5 µm. b‐d) Fluorescence images of the DAE/AgNW; before (b), and after the Write laser (23.9 GW/cm^2^, 1 s) (c), then the Erase laser (2.69 GW/cm^2^, 1 min) irradiation (d).

AgNWs were next embedded in an amorphous layer of DAE, 1,2‐bis(2‐ethyl‐6‐phenyl‐1‐benzothiophene‐1,1‐dioxide‐3‐yl)perfluoro cyclopentene (see SI, Section 1), the open form has absorption below 400 nm and is non‐fluorescent. It converts to the closed form upon UV excitation, absorbing visible light (400–520 nm) and emitting fluorescence at ≈550 nm (**Figure** **3**). The closed form reverts to the open form with visible photoexcitation. The equilibrium between the isomer forms is thus easily estimated by fluorescence. The as‐prepared DAE‐coated AgNWs exhibited a mixture of both DAE forms. Therefore, they were initially irradiated with intense 488 nm wide‐field illumination to completely switch the photochrome to the non‐fluorescent open form before experiments. Fluorescence images in **Figure** **4** were collected with 0.95 W/cm^2^ wide‐field 488 nm excitation, which hardly triggered photochromism. “Write” laser (820 nm, 120 fs, p‐polarized, 23.9 GW/cm^2^ for 1 s) was then focused at the left end of the DAE‐coated AgNW, launching propagating SPPs and inducing cyclization reaction as indicated by fluorescence recovery along the AgNW (Figure 4c). In our experiments, the Write laser was typically focused on the left end of AgNWs for consistency in data presentation. However, we experimentally verified that excitation from either the left or right ends of AgNWs results in comparable fluorescence recovery and plasmon propagation behavior under identical conditions. This excitation symmetry was also confirmed in vertically oriented AgNWs with a vertically polarized Write laser, where excitation from the top or bottom ends yielded equivalent results. These observations indicate that the plasmonic response is symmetric concerning the wire axis, provided that the polarization, wavelength, and other excitation parameters are kept constant. The fluorescence recovery reached a plateau within ≈0.2 s of irradiation (Figure S6, Supporting Information). As a control, the Write laser was applied with polarization perpendicular to the longitudinal axis of the AgNW (defined as s‐polarized, Figure S1, Supporting Information), which barely couples with SPPs at the ends of AgNW (Figure S7, Supporting Information). The fluorescence recovery was then only observed at the excited end of the AgNW (Figure S8, Supporting Information), confirming that the fluorescence recovery was induced by propagating SPPs on the AgNW.

**Figure 5:**
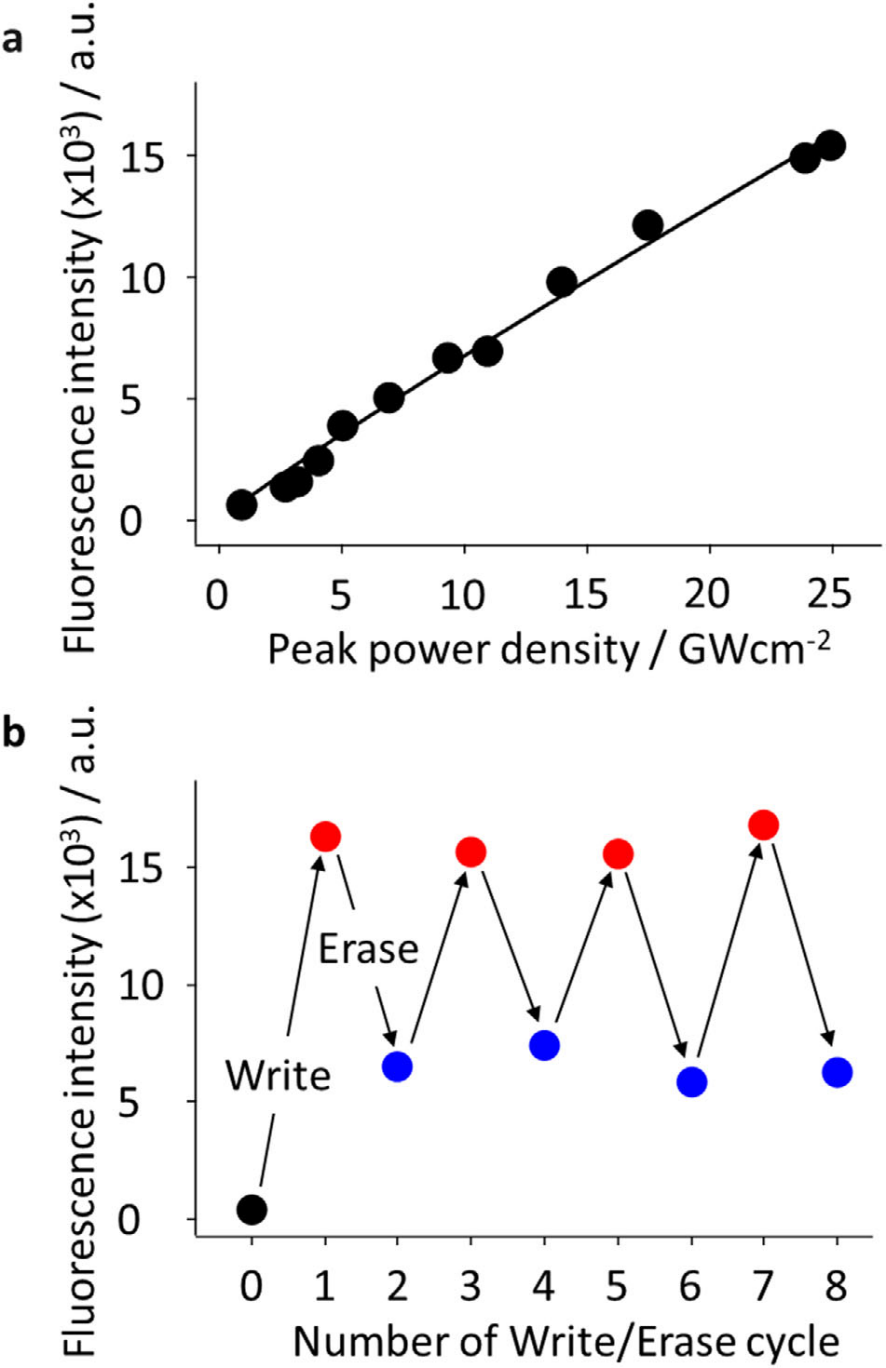
Controlling DAE PSS using single‐color SPPs. a) Excitation power dependence of fluorescence recovery. b) DAE fluorescence intensity after sequential Write/Erase laser irradiation.

The absorption of both open and closed forms of DAE is negligible at 820 nm. Thus, propagating SPP‐induced multiphoton excitation must occur along the AgNW as discussed on the two‐photon excitation spectra of Rh6G. However, DAE fluorescence recovery was found to be linearly proportional to the Write laser power (**Figure** **5**a). This linearity is the overall result of propagating SPP‐induced three‐photon cyclization and two‐photon cycloreversion reactions. These reactions switch between the DAE forms and a photostationary state (PSS) is attained (see Section S9, Supporting Information).^[^ ^22^ ^]^ The different power dependences of these reactions open the possibility of intensity‐tuning the DAE PSS using a single‐color NIR laser. Figure 4d is a wide‐field fluorescence image of the AgNW after “Erase” laser (820 nm, 120 fs, p‐polarized, 2.69 GW/cm^2^ for 1 min) was again focused on the left wire end, but with lower laser power. Quenching of the DAE fluorescence was observed along the AgNW, indicating that the DAE PSS shifted back toward the non‐fluorescent open form. At lower excitation power, the three‐photon‐induced cyclization reaction is effectively suppressed, allowing the two‐photon cycloreversion process to dominate during the Erase step. Although the quantum yield of cyclization is typically higher than that of cycloreversion in one‐photon processes,^[^ ^18^, ^23^ ^]^ multi‐photon excitation alters this relationship,^[^ ^24^ ^]^ enabling a more balanced contribution from both reactions. Nevertheless, the Erase step still requires significantly longer exposure, approximately 100 times that of the Write step (Figure S5B, Supporting Information), due to the lower overall efficiency of the ring‐opening process under the current excitation conditions. Furthermore, sequential fluorescence recovery/quenching was repeatedly performed by adjusting the NIR laser power (Figure 5b).

It should be noted that the fluorescence signals do not fully return to their initial values after a single Write/Erase switching cycle. This behavior originates from the photochemical nature of the switching mechanism. Prior to each experiment, the sample is pre‐irradiated with strong 488 nm light to convert nearly all DAE molecules to the open form, thus establishing a well‐defined and reproducible initial state. During the switching cycle, however, the same wavelength of fs pulsed laser is used for both the Write (ring‐closing) and Erase (ring‐opening) steps, relying on nonlinear multiphoton absorption. While the Erase step is primarily driven by two‐photon‐induced cycloreversion, the same beam can also induce a small amount of undesired three‐photon cyclization. This results in a dynamic competition between ring‐opening and ring‐closing processes, ultimately driving the system toward a PSS under continuous illumination. As a consequence, complete recovery to the original fluorescence signal level is not achievable under the current one‐color excitation scheme.

### 2.3 All‐Plasmonic Nanowire Memory Function

**Figure 6:**
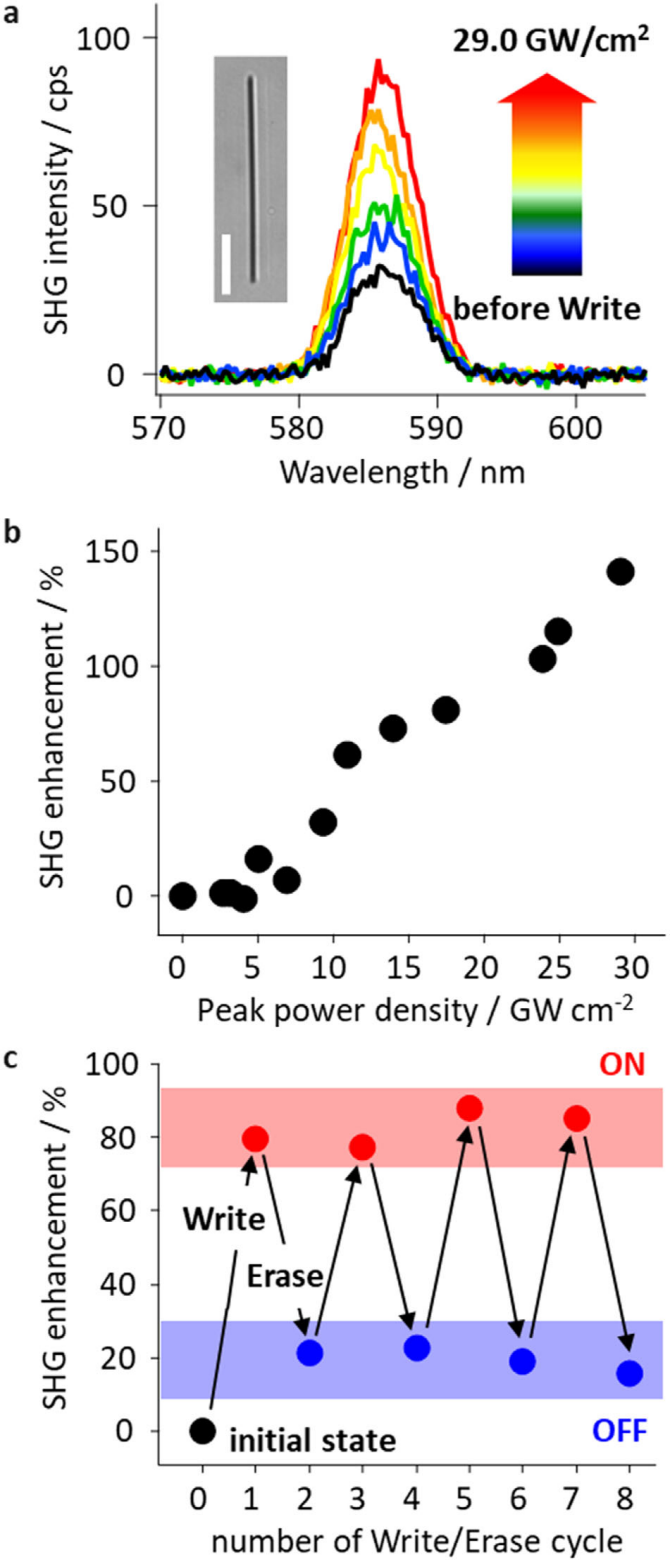
Control of SPP propagating efficiency. a) Spectra of the plasmonic SHG before and after Write laser irradiations. The inset is a transmission image of the AgNW used in this experiment. The scale bar is 2.5 µm. b) Write laser power dependence of the SHG enhancement. c) SHG enhancement over sequential Write/Erase cycles. The red/blue bands represent the ON/OFF states for the binary operation.

In a series of experiments, we explored how the SPP propagation efficiency along AgNW can be modulated by tuning the DAE PSS (Figure 1). The closed form of DAE, due to its delocalized π‐electrons, exhibits a higher refractive index.^[^ ^18^ ^]^ Since SPP propagation is highly sensitive to the refractive index of the surrounding medium, an increased refractive index leads to longer SPP propagation distances.^[^ ^25^ ^]^ This relationship allows modulation of SPP efficiency through adjustments to the DAE PSS. To monitor SPP propagation efficiency, we employed a “Readout” laser (1164 nm, 200 fs, p‐polarized, 208 MW/cm^2^). This laser, with its weak power and long wavelength, can excite the plasmonic SHG^[^ ^19^ ^]^ at the distal wire end without significantly inducing multiphoton photochromism or altering the DAE PSS (Figure S9, Supporting Information). Before and after exposure to the Write laser, the plasmonic SHG signals at 582 nm were recorded (**Figure** **6**a). The Write laser irradiation increased the SHG signals up to an SHG enhancement of 150%, given by ∼ ($I_{SHG}^{a}-I_{SHG}^{b}$)/$I_{SHG}^{b}$×100, where $I_{SHG}^{b}$ and $I_{SHG}^{a}$ are SHG intensity measured before and after Write laser irradiation, respectively. SHG enhancement was unchanged between Write/Erase events during the Readout (Figure S10, Supporting Information), demonstrating the thermal stability of the DAE molecules. SHG enhancement also depended on AgNW length (Figure S11, Supporting Information), consistent with the plasmonic SHG induced by SPP propagation. While the SHG enhancement was generally consistent among AgNWs of similar length, a variation of several to tens of percent was observed. This variability likely arises from experimental factors such as laser intensity fluctuations, slight differences in AgNW apex morphology, and minor misalignments during focusing. Despite these factors, the overall trend of SHG dependence on AgNW length remains robust. Improvements in laser stability and alignment reproducibility will be pursued to further minimize measurement variability.

Figure 6b shows a linear relationship between SHG enhancement and Write laser power, confirming that SPP propagation efficiency is controlled by the DAE PSS as linearity was seen in the DAE fluorescence recovery. Sequential binary modulation was further demonstrated (Figure 6c), with the Write laser inducing a ≈90% enhancement (defined as ON‐state) and the Erase laser reducing SHG to ≈20% (defined as OFF‐state). Repeated Write/Erase cycles modulated SPP propagation between ON/OFF states, analogous to a memory having binary resistance levels (ON/OFF states), where SPP propagation efficiency reflects the “resistance” based on the propagation history and the resistance can be read as plasmonic SHG intensity.

Nonlinear plasmonics^[^ ^26^ ^]^ offers significant advantages in this system. Write/Erase functions could be performed in the one‐photon regime, i.e., by focusing two lasers, in the UV and Vis, to induce DAE cyclization and cycloreversion, respectively. Using herein the nonlinear regime with intensity modulation of a one‐color NIR laser to write and erase the propagating SPP levels potentially reduces the system complexity. Moreover, multiphoton excitation can significantly enhance the quantum yield of DAE cycloreversion, increasing it by up to tenfold compared to one‐photon processes.^[^ ^24^ ^]^ This enhancement reduces the dominance of the ring‐closing reaction typically seen under one‐photon excitation. As a result, the PSS can be achieved under the applied intensity range and more rapidly, with both ring‐opening and ring‐closing reactions contributing more equally to the system's equilibrium. NIR excitation also supports longer SPP propagation due to reduced Ohmic damping and the facilitation of long‐range SPP modes (Figure S2, Supporting Information), enhancing flexibility for designing optical circuits.

The described all‐plasmonic nanowire memory does not require any additional architecture to modify the level of SPP propagation, but the modulation is achieved by SPPs themself propagating along the nanowire. Thanks to the simpleness of the self‐modulation feature, the all‐plasmonic nanowire memory would be a promising element in plasmonic integrated circuits. AgNWs have been proven as an information carrier path, ruler, logic gate, etc.^[^ ^27^ ^]^ AgNW manipulation techniques^[^ ^28^, ^29^, ^30^, ^31^ ^]^ would be key to implementing the nanowire memory in the plasmonic integrated circuit. Furthermore, a randomly interconnecting AgNW network^[^ ^32^, ^33^ ^]^ is potentially utilized as an imitation system of a neuron network,^[^ ^34^ ^]^ in which the memories and processing units can be overlapped and interconnected, solving the von Neumann bottleneck.

In the context of achieving high‐density memory arrays, spatial resolution and potential cross‐talk between closely packed nanowire units are important issues that must be addressed. While the present study focuses on demonstrating the fundamental operations (Write, Erase, and Readout) at the single‐object level, our results also provide insight into the feasibility of selective excitation within sub‐diffraction‐limited proximities. Although the distance between two AgNW apexes in our current experiments (∼681 nm) is slightly above the diffraction limit of our system (≈671 nm), we observed significantly different fluorescence recovery depending on the orientation of the AgNWs and the polarization of the excitation light. By slightly adjusting the polarization and alignment of the excitation beam, selective excitation, and fluorescence recovery were achieved on either AgNW1 or AgNW2, demonstrating orientation‐based optical addressability even within near‐diffraction‐limited spacing (Figure S12, Supporting Information).

These results suggest that, in principle, excitation below the diffraction limit is possible by exploiting the strong polarization dependence of propagating SPPs in AgNWs. Particularly, p‐polarized light strongly excites SPPs along the long axis of the AgNW, enabling super‐resolution operations such as Write, Erase, and Readout based on AgNW orientation or the structure AgNW apex. Furthermore, future integration of plasmonic concentration techniques^[^ ^35^, ^36^ ^]^ and near‐field excitation methods^[^ ^37^, ^38^ ^]^ could enable even finer spatial resolution, allowing selective access to individual nanowires placed at sub‐diffraction‐limit distances without cross‐talk.

To realize this potential, the development of high‐precision alignment and placement techniques for AgNWs will be essential. While this presents a technical challenge, it also opens new avenues for scaling up all‐plasmonic memory systems toward practical high‐density, low‐crosstalk integrated devices. In addition, although this study focuses on AgNWs as plasmonic waveguides, the underlying principle of orientation‐selective excitation and self‐modulation by propagating SPPs could, in principle, be extended to other lithographically defined plasmonic waveguides with precisely engineered geometries. Such an approach may offer even greater scalability and integration potential in future plasmonic memory architectures.

## 3 Conclusion

In summary, we have provided experimental proof‐of‐concept of all‐plasmonic memory operation using sub‐diffraction‐limit plasmonic waveguides, photochromic DAEs, and NLO. This study explored the plasmonic waveguide effect on AgNWs, from a linear to a nonlinear regime, to demonstrate one‐color reversible photochromic reactions in a DAE derivative via nonlinear remote excitation. Applying this reduces the excitation volume to sub‐diffraction‐limit dimensions and provides a unique platform for the control of photochemical reactions along AgNWs. The Write/Erase functions in the nanowire memory modify the SPP propagation efficiency (the resistance state). This is achieved by tuning the DAE PSS, which defines the refractive index surrounding AgNWs, and thus the plasmon propagation efficiency. By interacting with propagating SPPs, DAE molecules undergo both three‐photon cycloreversion and two‐photon cyclization reactions, resulting in a PSS shift. The switching Write/Erase functions is done by simply adjusting the power of the one‐color NIR laser. Furthermore, plasmonic SHG readouts the resistance states. The SHG is found to be modified only when the Write/Erase laser is applied thanks to the thermal stability of DAE. This concept could be generalized to a wide variety of plasmonic waveguides (metallic stripes,^[^ ^39^, ^40^ ^]^ nanoparticle arrays,^[^ ^41^, ^42^ ^]^ grooves/slots/teeth,^[^ ^43^, ^44^, ^45^ ^]^ tapered plasmonic waveguides,^[^ ^46^, ^47^ ^]^ etc.) and photo‐responsive materials (phase change materials,^[^ ^2^ ^]^ and photo‐polymerizing monomers,^[^ ^48^ ^]^ etc.), encouraging subsequent development of all‐plasmonic integrated circuits with memory function with potential to solve the von Neumann bottleneck. The challenge of integration of all‐plasmonic memories into ultra‐low footprint optical devices awaits next.

## 4 Experimental Section

### AgNW Synthesis

The AgNWs were fabricated using the polyol method, following a report.^[^ ^49^ ^]^ Briefly, 5 mL of 0.15 M polyvinylpyrrolidone (Mw ≈ 40000) solution in ethylene glycol (EG) was heated at 160 °C for 60 min. Then, 40 µL of CuCl_2_ (4 × 10^−3^ M) solution in EG was injected. After 10 min, 2.5 mL of an AgNO_3_ EG solution (0.12 m) was added dropwise, at an injection rate of 100 µL min^−1^, under magnetic stirring (600 rpm). The mixture was maintained at 160 °C for another 1 h, which produced a high yield of AgNWs. The AgNWs were then washed three times with isopropanol (IPA). The average diameter of the AgNWs was 150 nm, and their lengths were typically longer than 10 µm.

### Fluorescent Diarylethene Synthesis

All reagents used in the synthesis were obtained commercially and used without further purification, unless otherwise specified. The ^1^H nuclear magnetic resonance (NMR) and ^13^C NMR spectra were recorded using tetramethylsilane as the internal standard on a JEOL JNM‐EX400 at 400 and 100 MHz, respectively. Mass spectra were determined using a Bruker Autoflex Speed mass spectrometer. The purity of the final synthesized diarylethene compound was ≥98% as determined by high‐performance liquid chromatography (HPLC) with a Wokosil‐5SIL column (4.6 × 250 mm). The diarylethene compound used in this work, 1,2‐bis(2‐ethyl‐6‐phenyl‐1‐benzothiophen‐1,1‐dioxide‐3‐yl)perfluorocyclopentene, was synthesized according to the reported procedure.^[^ ^23^ ^]^ The structure and purity of the synthesized DAE were confirmed by ^1^H and ^13^C NMR, high‐resolution mass spectroscopy, and UV‐Vis absorption spectroscopy, all of which matched well with previously reported data.

### Sample Preparation

The AgNW IPA of 30 µL solution was spin‐coated onto cleaned glass substrates, then the substrates were heated on a hotplate at 80 °C for 30 min. Thereafter, 30 µL of a Rh6G and polyvinylalcohol (PVA) solution in MeOH (0.5 mm for Rh6G and 0.5 wt.% for PVA), or a DAE solution in IPA (1 mM) was spin‐coated onto the substrates for two‐photon excitation spectrum measurement and demonstration of all‐plasmonic AgNW optical memory function, respectively.

### Two‐Photon Excitation Spectrum Measurement

The experiment was conducted using an inverted optical microscope (Ti‐U, Nikon) equipped with a piezoelectric stage (P517.3CL, Physik Instrument). The experimental setups are shown in Figure S1A (Supporting Information). Briefly, a fs laser beam (Spectra‐Physics, Mai Tai, 750–910 nm, 120 fs, 80 MHz) was divided into two beams by a 90:10 beamsplitter. The weaker beam was guided into the microscope directly to irradiate a AgNW. Another beam was guided into an optical parametric oscillator (OPO) (Spectra‐Physics, Inspire HF 100) for energy conversion. The output from OPO (890‐1100 nm) was guided into the microscope. The both beams were polarized parallel to the longitudinal axis of a AgNW defined as p‐polarized (as schematically presented in Figure S1B, Supporting Information), and focused on the sample through an objective lens (Nikon, 100x, oil immersion, N.A. 1.49). The laser power was kept at about 150 µW for two‐photon excitation on AgNW. The same objective lens was used to collect fluorescence signals. Fluorescence spectra were recorded using a charge‐coupled device (CCD) camera (DU920P, Andor) at an operating temperature of −85 °C equipped with a spectrograph (iHR320, Horiba). A shortpass filter (ET800SP‐2P, Chroma or FES0700, Thorlabs) and a pinhole (100 µm diameter) were positioned in front of the entrance to the spectrograph to cutoff the excitation laser. Spatial resolved fluorescence spectrum measurements were achieved with direct and remote excitation configurations. Those systems are described in more detail in the SI section 4.

### One‐Color Multiphoton Photochromic Reactions on AgNW

The experiment was conducted on the same inverted optical microscope setup as for the excitation spectrum measurements. Propagating SPPs on AgNWs, and associated multiphoton photochromic reactions, were excited with the same fs pulsed laser beam (820 nm, 120 fs, 80 MHz) focusing on one end of a AgNW. The laser power was tuned to provide the optical memory Write and Erase beams. The irradiation time was controlled by using a mechanical shutter (Newport). Fluorescence images were captured by imaging electron‐multiplying CCD (Hamamatsu Photonics, ImagEM X2) at an operating temperature of −35 °C and with wide‐field 488 nm laser excitation (Stabilite 2018, Spectra‐Physics). Widefield illumination was achieved by inserting an additional lens (f = 500 mm) before the objective. A bandpass filter (Chroma) was positioned in front of the camera to eliminate excitation laser light.

### Remote Excitation SHG (Plasmonic SHG)

The plasmonic SHG measurement was conducted on the same inverted optical microscope setup. The method has been described elsewhere.^[^ ^19^ ^]^ The Readout laser (1164 nm, 200 fs, 80 MHz) was obtained from the same OPO and focused on one end of a AgNW, inducing the remote excitation SHG at the distal end (plasmonic SHG). The optical configuration for the remote excitation SHG is described in Section S2 (Supporting Information). The detected SHG signals were recorded using the same CCD camera and spectrograph. A shortpass filter (ET800SP‐2P, Chroma) and a pinhole (100 µm diameter) were positioned in front of the entrance to the spectrograph.

### Dark‐Field Measurements

Dark‐field (DF) measurements were conducted using the same inverted optical microscope. A halogen lamp (12 V/100 W halogen lamp, Nikon) was used as the light source. The light of the lamp was focused through a DF condenser (TI‐DF, dry, N.A. 0.95‐0.80). Scattered light was collected by an objective lens (Plan Fluor ELWD 40x, N.A. 0.60, Nikon) and detected by either a color imaging CCD (Thorlabs) or the same confocal spectroscopic system used for two‐photon excitation spectrum measurement. The raw scattering spectra were divided by the lamp spectrum to obtain the corrected DF spectra.

## Conflict of Interest

The authors declare no conflict of interest.

## Supporting information



Supporting Information

## Data Availability

The data that support the findings of this study are available from the corresponding author upon reasonable request.;
