# Supplementary material for: A Sub‐Diffraction‐Limit Dimension All‐Plasmonic Optical Memory Using Non‐Linear Photochromism
Source: Adv Sci (Weinh). 2025 May 8;12(27):2502890. doi: 10.1002/advs.202502890 (PMC12279226; doi:10.1002/advs.202502890)
Supplement: Supplementary file 1 — Supporting Information [file ADVS-12-2502890-s001.docx]

Supporting Information

A Sub-diffraction Limit Dimension, All-plasmonic Optical Memory using Non-linear Photochromism

Shuichi Toyouchi,* Mathias Wolf, Kenji Hirai, Yasuhiko Fujita, Tomoko Inose, Beatrice Fortuni, Eduard Fron, Johan Hofkens, Steven De Feyter, James Hutchison, Tsuyoshi Fukaminato,* and Hiroshi Uji-i*

Contents

**§1. Experimental setup**

**§2. Excitation wavelength dependence of SPP propagation efficiency on AgNWs**

**§3. Two-photon excited fluorescence spectra of Rh6G on AgNW**

**§4. Optical configuration of remote excitation spectroscopy**

**§5. Excitation spectrum of Rhodamine 6G without AgNW**

**§6. Time scale of Write and Erase processes**

**§7. Dark-field imaging and spectroscopy**

**§8. Write laser polarization dependence of DAE fluorescence recovery on AgNWs**

**§9. Photostationary state kinetics**

**§10. Stability of the AgNW optical memory during Readout laser application**

**§11. AgNW length dependence of DAE/AgNW SHG enhancement**

**§12. Polarization-controlled selective fluorescence recovery on closely spaced AgNWs**

**§1. Experimental setup**


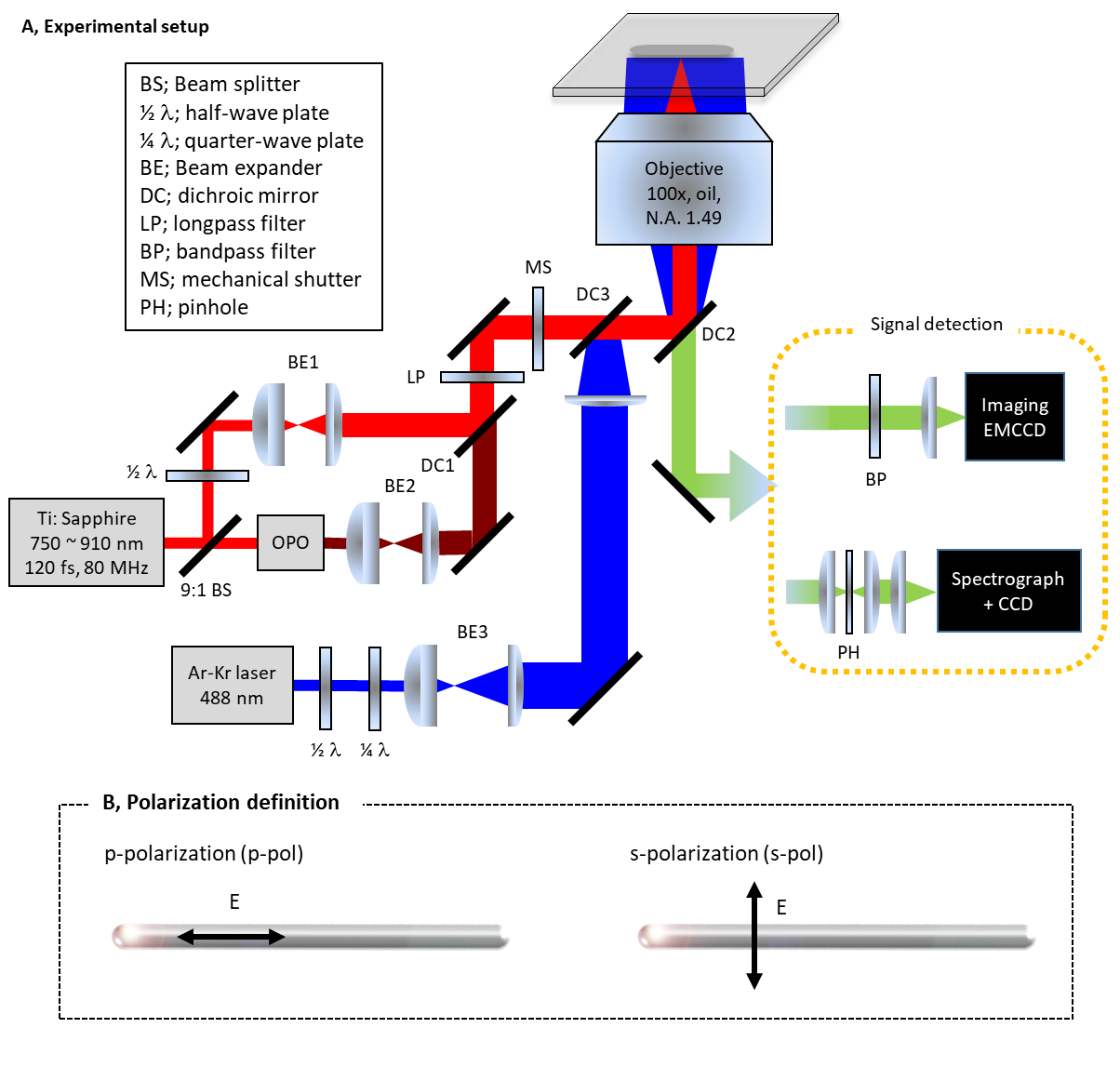


**Figure S1. Schematic of experimental apparatus.** (A) Schematic illustration of the experimental setup. BS; Beam splitter, ½ λ; half-wave plate, ¼ λ; quarter-wave plate, BE; Beam expander, DC; dichroic mirror, LP; longpass filter, BP; bandpass filter, MS; mechanical shutter, PH; pinhole. (B) Schematic illustration of the definition of p- and s-polarization light excitation used in this study.

**§2. Excitation wavelength dependence of SPP propagation efficiency on AgNWs**

Here the propagating SPP efficiencies along the AgNWs for various excitation wavelengths were examined by using fluorescence imaging.^[1]^ We embedded AgNWs having an average diameter of 150 nm in a PVA matrix doped with Rh6G and focused the excitation laser at the left end of AgNWs, launching propagating SPPs and inducing Rh6G fluorescence along the AgNWs. The propagating SPPs can be tracked by the Rh6G fluorescence. **Figure S2** shows optical transmission images (left upper), fluorescence images (left lower) and cross-sections of each fluorescence image along the AgNWs long axis (right) for 410 nm fs pulsed laser (Figure S2A), 488 nm CW laser (Figure S2B), 532 nm CW laser (Figure S2C), 820 nm fs pulsed laser (Figure S2D) and 1164 nm fs pulsed laser (Figure S2E). The cross-sections were analyzed assuming single exponential decay exp(-x/τ), where τ is the 1/e decay length. Considering that Rh6G at the left end was excited not only by SPPs (near-fields) but also laser excitation light (far-field), the curve fitting was conducted for data points from at least 2 μm away from the left end to reduce the contribution of the latter (the red lines plotted in each cross-section shows the region over which the fits were made). The determined fluorescence decay lengths for 410 nm, 488 nm, 532 nm, 820 nm and 1164 nm excitation are 0.81 ± 0.14 μm (average of 5 AgNWs), 1.18 ± 0.27 μm (average of 5 AgNWs), 1.33 ± 0.26 μm (average of 5 AgNWs), 6.03 ± 1.98 μm (average of 16 AgNWs) and 10.39 ± 4.21 μm (average of 6 AgNWs), respectively. Considering that Rh6G was excited via two-photon excitation with 820 nm and 1164 nm, the SPP decays for 820 nm and 1164 nm were doubled, obtaining 12.1 ± 4.0 μm and 20.8 ± 8.4 μm, respectively (summarized in Figure S2E). The SPP decay increases with increasing excitation wavelength, consistent with previous reports^[1, 2]^ due to reduced Ohmic dumping and different SPP modes being excited along the AgNW.


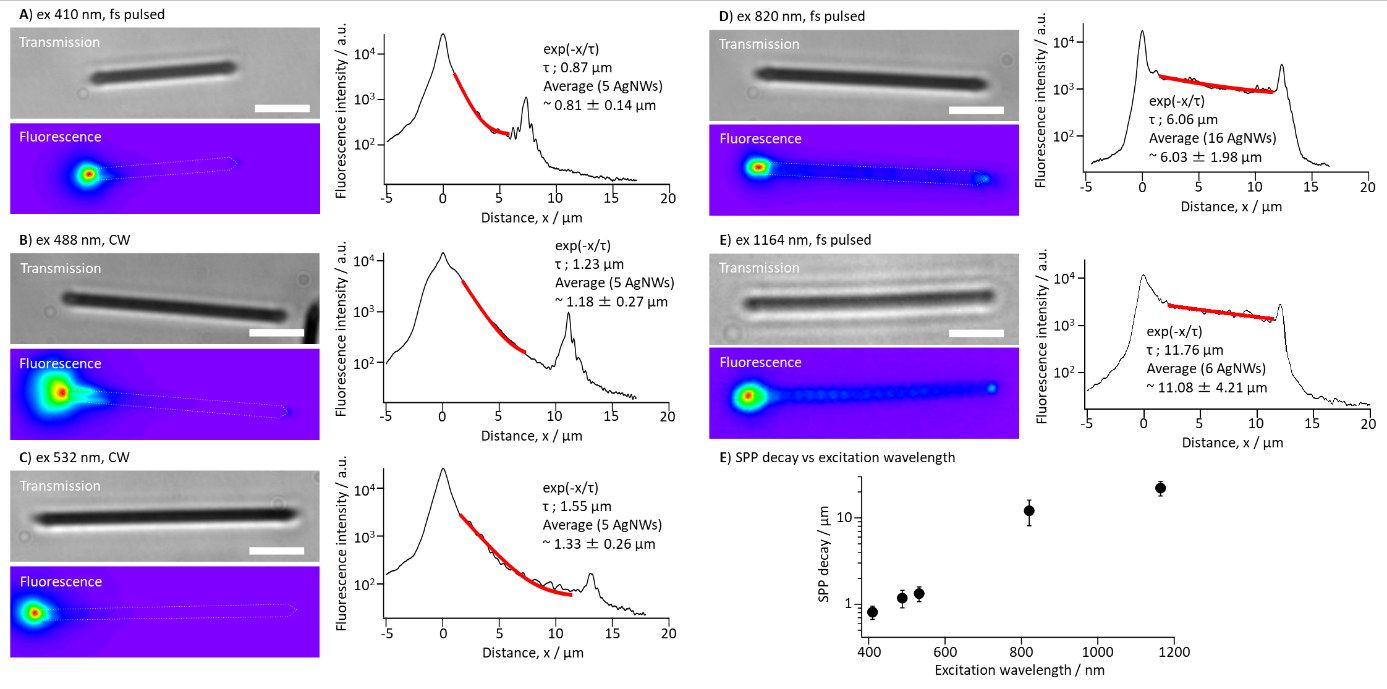


**Figure S2. Excitation wavelength dependence of SPP propagation efficiency on AgNWs.** Transmission (left upper) and fluorescence (left lower) images of a AgNW embedded in a Rh6G/PVA layer, and cross-sections of each fluorescence image along the AgNW long axis (right). Excitation light sources for fluorescence are 410 nm fs pulsed laser (A), 488 nm CW laser (B), 532 nm CW laser (C), 820 nm fs pulsed laser (D), and 1164 nm fs pulsed laser (E). All lasers were focused on the left wire end to launch propagating SPPs. The propagation can be tracked by Rh6G fluorescence. The scale bar is 2.5 μm. White dashed lines are guides for the eye indicating AgNWs.

**§3. Two-photon excited fluorescence spectra of Rh6G on AgNW**

Fluorescence spectra of Rh6G detected locally at the left end (“in”), the distal end (“out”), and along the AgNW body, are shown in **Figure S3**a. For the fluorescence measurements at the distal end and along the AgNW body, so-called “remote excitation” described in SI, Section 4, was employed. Regardless of the detection position, the excitation peak power density dependence on the Rh6G fluorescence intensities displays a quadratic power dependence (Figure S3b), indicating that Rh6G molecules were excited through two-photon processes all over the AgNW.


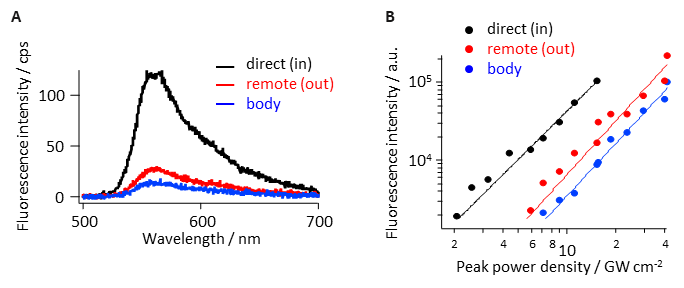


**Figure S3. Two-photon excited fluorescence spectra of Rh6G on AgNW.** (A) Spatially-resolved Rh6G fluorescence spectra were taken at the left end (black line, direct excitation (in)), the distal end (red line, remote excitation (out)), and the body part (blue line) of the AgNW. (B) Excitation peak power density dependence of the Rh6G fluorescence intensities detected at the left end (black line, direct (in)), the distal end (red line, remote (out)), and the body part (blue line) of the AgNW.

**§4. Optical configuration of remote excitation spectroscopy**

The excitation and detection scheme is displayed in **Figure S4**, in which the excitation laser light and fluorescence or SHG signals are highlighted with a red thick line and a green thin line, respectively. Direct excitation is achieved by focusing the excitation laser at an arbitrary position on a AgNW with an objective (e.g. at the left wire end in Figure S4), and fluorescence or SHG signals from the same end are collected through the same objective. The collected signals pass through a pinhole (diameter 100 μm), and then are guided into a spectrograph. For remote excitation, the excitation laser is displaced from the optical axis by mirrors before the microscope (deliberate misalignment), while the pinhole position in the detection path is still adjusted to detect a signal from the optical axis. In this way, excitation light can be focused at the right end of a AgNW while only fluorescence or SHG signals generated at the left wire end pass through the pinhole and are measured. In the remote excitation configuration, confocality ensures that fluorescence signals at the detection point are free of directly excited fluorescence signals when the excitation and detection points are separated by >2 microns.


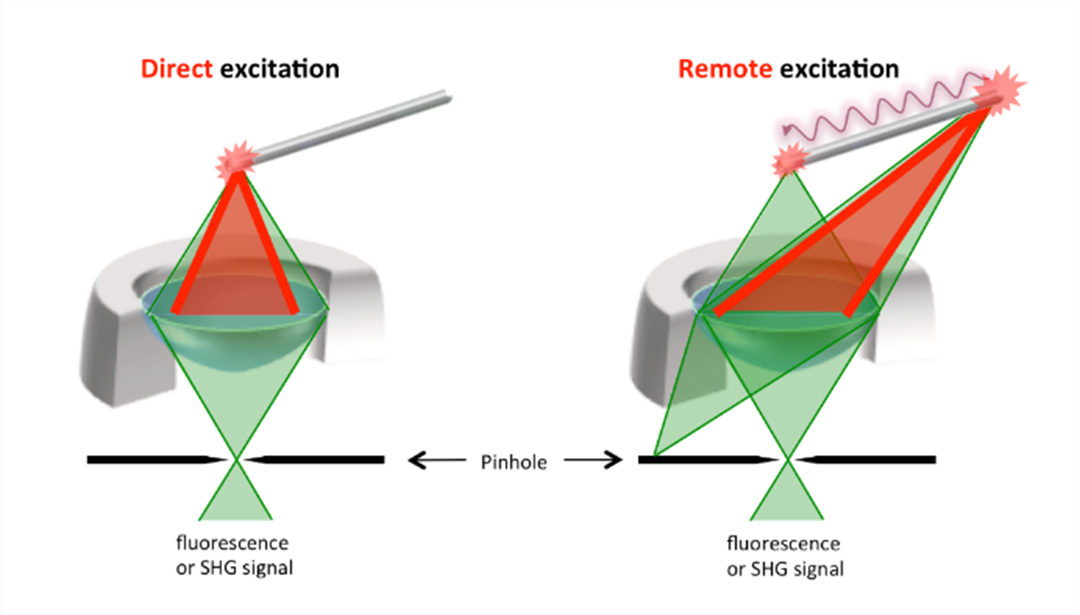


**Figure S4. Optical configurations for direct excitation and remote excitation spectroscopy.**

**§5. Excitation spectrum of Rhodamine 6G without AgNW**

One-photon and two-photon excitation spectra of Rh6G in a PVA film without AgNWs were recorded. This measurement was conducted with the same optical microscope system as already described for the Rh6G/AgNW experiments. One-photon excitation was done with 400 ~ 550 nm fs pulsed laser obtained from the same OPO (see Section 1). The laser power was kept at about 50 nW and 500 μW for one-photon and two-photon excitation, respectively. The obtained one-photon and two-photon excitation spectra are shown in **Figure S5** (black and red circles respectively). The one-photon excitation spectrum has a single main peak around 540 nm and shows a clear mirror image with the fluorescence spectrum, while the two-photon excitation spectrum has two peaks around 480 nm and 400 nm. This is because Rh6G possesses approximately C_2v_ symmetry in the ground state.^[3]^ Briefly, the S_0_ ground state of the π-electrons belongs to the totally symmetric representation A_1_, whereas the excited states S_1_ and S_2_ belong to B_2_ and A_1_, respectively. The S_0_→S_1_ transition of the Rh6G peak at around 540 nm is permitted for one-photon absorption, while the S_0_→S_2_ transition peak at around 400 nm is symmetry forbidden. Consequently, in the one-photon excitation spectrum, the S_0_→S_1_ transition is predominantly observed (Figure S5, red circle). However, for the two-photon absorption, both S_0_→S_1_ and S_0_→S_2_ transitions are symmetry allowed, although the S_0_→S_2_ transition is generally enhanced.^[3]^ Therefore, the S_0_→S_2_ transition is observed predominantly in the two-photon excitation spectrum without the AgNW.


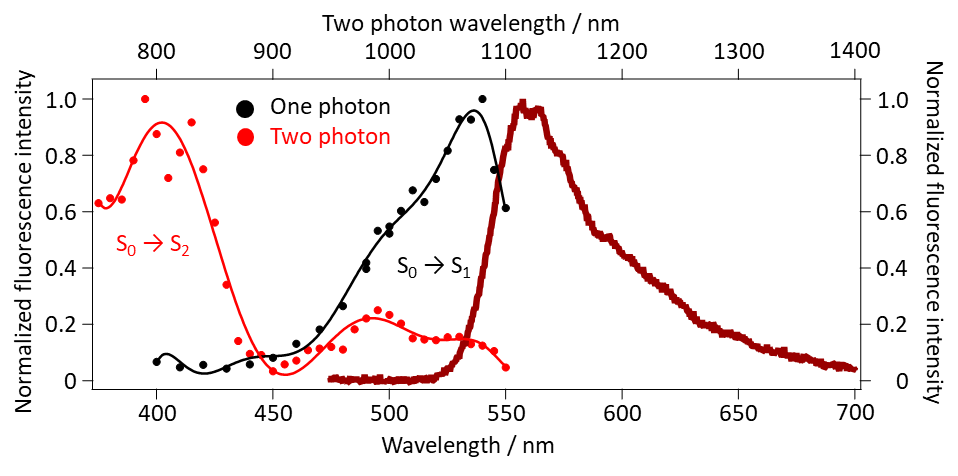


**Figure S5. One-photon and two-photon excitation spectra of Rh6G in PVP measured without AgNW.** Black closed circles and red closed circles represent one-photon and two-photon excitation spectral intensities, respectively, black and red solid lines are guides for the eye for one-photon and two-photon excitation, respectively. The Rh6G two-photon excitation fluorescence spectrum excited at 820 nm is also included (dark red, thick line).

**§6. Time scale of Write and Erase processes**

To examine the time scales of Write and Erase processes, the fluorescence recovery (Write) and bleaching (Erase) of the DAE along the AgNWs were measured after Write (820 nm, 120 fs, 80 MHz, p-polarized, 23.9 GW/cm^2^) and Erase laser (same as the Write laser, but 2.69 GW/cm^2^) irradiation with various irradiation times. The irradiation time was controlled by using a mechanical shutter (see Section 1). For the Write timescale measurement, in advance of each Write laser irradiation, the DAE was irradiated by intense 488 nm light wide-field illumination (3 mW for 10 min) to switch it completely to the non-fluorescent open form. The fluorescence recovery, defined as the difference in fluorescence intensity before and after the Write laser irradiation, is plotted in **Figure S6**A. The fluorescence recovery reached a plateau within ~ 0.2 s of irradiation. Therefore, the photostationary state (PSS) between open and closed forms under Write laser irradiation is attained on sub-second timescales. For the Erase process, in advance of each Erase laser irradiation, the DAE was again irradiated by the same 488 nm illumination and sequential Write laser (23.9 GW/cm^2^, 1 s) to switch the photochrome to the fluorescent closed form along the whole AgNW. The fluorescence bleaching, also defined as the difference in fluorescence intensity before and after the Erase laser irradiation, is plotted in Figure S6B. The fluorescence bleaching reached a plateau after around 1 min of irradiation (Figure S6B), indicating that the Erase process takes 100 times longer than the Write process. This timescale difference can be explained by differences in the reaction quantum yields for cyclization and cycloreversion. Typically, the quantum yield of cyclization is several orders of magnitude larger than that of cycloreversion under one-photon excitation.^[4, 5]^ However, in the case of multi-photon excitation, this relationship can change. The quantum yields of both cyclization (ring-closing) and cycloreversion (ring-opening) reactions become more comparable, as higher-order absorption processes contribute differently to each reaction. In fact, it has been reported that under multi-photon excitation, the quantum yield of the ring-opening reaction increases significantly.^[6]^ As a result, under multi-photon excitation, the reaction balance shifts, leading to a more balanced contribution from both reactions, which can alter the steady-state population distribution (PSS) compared to one-photon excitation.


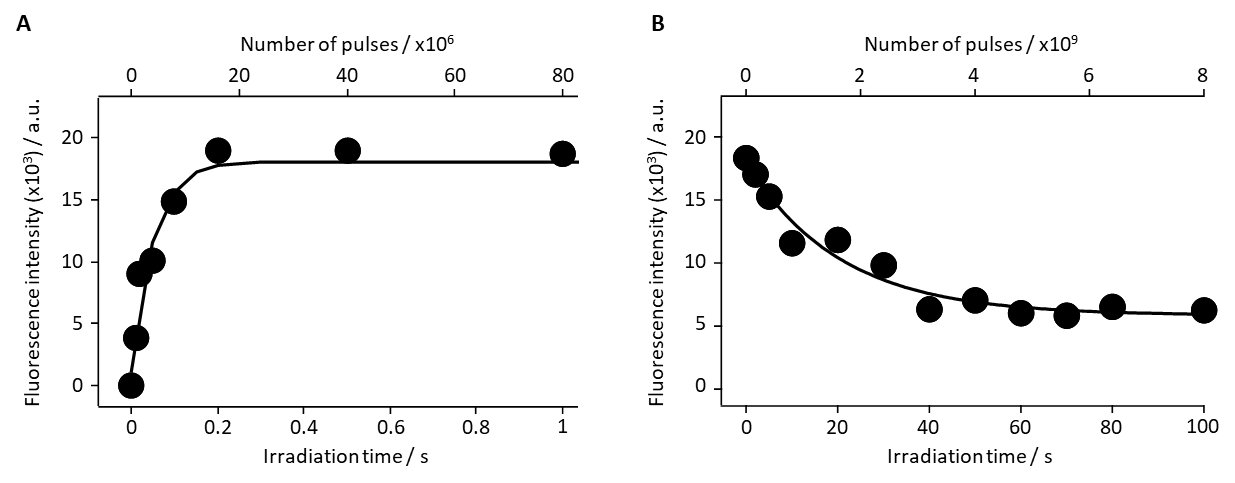


**Figure S6. Time-course fluorescence recovery.** DAE/AgNW fluorescence recovery plotted as a function of irradiation time of Write laser (820 nm, 120 fs, 80 MHz, p-polarized, 23.9 GW/cm^2^) (A) and Erase laser (same as the Write laser but 2.69 GW/cm^2^) (B). Black solid lines are guides to the eye.

**§7. Dark-field imaging and spectroscopy**

DF imaging/spectroscopy was performed for AgNWs. **Figure S7**A to C are DF images of a AgNW obtained under DF illumination. The illumination was non-polarized (Figure S7A), s-polarized (Figure S7B) and p-polarized light (Figure S7C). Non-polarized and s-polarized light illumination was scattered almost uniformly along the whole AgNW, while p-polarized light was scattered all along the AgNW but slightly more efficiently at the ends of the AgNW, indicating that p-polarized light couples with SPPs more efficiently at the ends of AgNWs. Nevertheless, the scattering at the AgNW ends is difficult to measure as it can be overwhelmed by scattering from the body part of the AgNWs. To address this issue, the dark-field illumination spot was displaced from the AgNW.^[7]^ With such displaced illumination, the obtained dark-field image shows bright spots only at the ends of the AgNW (Figure S7D). Figure S7E and F show DF spectra measured at the left end of the AgNW, when illumination was placed on the AgNW and displaced from the AgNW, respectively. With normal (direct) illumination, the DF spectrum with s-polarized light exhibits higher scattering intensity than it does with p-polarized light (Figure S7E), due to the aforementioned overwhelming of the wire-end DF scattering by scattering from the body part of the wire. On the other hand, with the displaced illumination (Figure S7F), the DF spectrum with p-polarized light exhibits higher scattering intensity, proving that p-polarized light couples with SPPs at the ends of the AgNWs more efficiently.


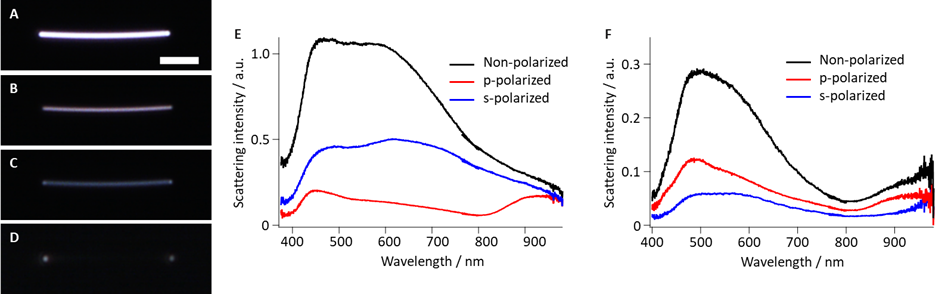


**Figure S7. Dark-field images and spectra of AgNW.** (A-D) Dark-field images of a AgNW embedded in a PVP film obtained with dark-field illumination directly on the AgNW, with non-polarized (A), s-polarized (B) and p-polarized light (C), and obtained when the dark-field illumination was displaced from the AgNW with non-polarized light (D). The scale bar is 5 μm. (E, F) Dark-field spectra measured at the left end of the AgNW obtained when the dark-field illumination was directly on the AgNW (E) and displaced from the AgNW (F).

**§8. Write laser polarization dependence of DAE fluorescence recovery on AgNWs**

For a deeper understanding of the DAE fluorescence recovery on AgNWs, the Write laser polarization dependence was examined. **Figure S8**A shows a DAE/AgNW used in this experiment. Before the experiments, the DAE was switched to the non-fluorescent open form as described in Section 1. The fluorescence image of the DAE/AgNW exhibits negligible levels of fluorescence (Figure S8B). The Write laser (820 nm, 120 fs, 80 MHz, p-polarized, 23.9 GW/cm^2^ for 1 s) was irradiated at the left end of the DAE/AgNW, inducing fluorescence recovery along the whole AgNW (Figure S8C). On the other hand, the same Write laser but with s-polarization induced significantly lower fluorescence recovery (only few % compared with p-polarization) along the whole AgNW (Figure S8D). The polarization dependence of the fluorescence recovery can be explained by the inefficient coupling of s-polarized light with surface plasmons at the ends of AgNWs due to the momentum matching condition. P-polarized light couples with SPPs more efficiently and launches propagating SPPs (see Section 7). This polarization dependence is further proof that the shift of the PSS of DAE, as monitored by fluorescence recovery, was induced by the propagating SPPs along the AgNW.


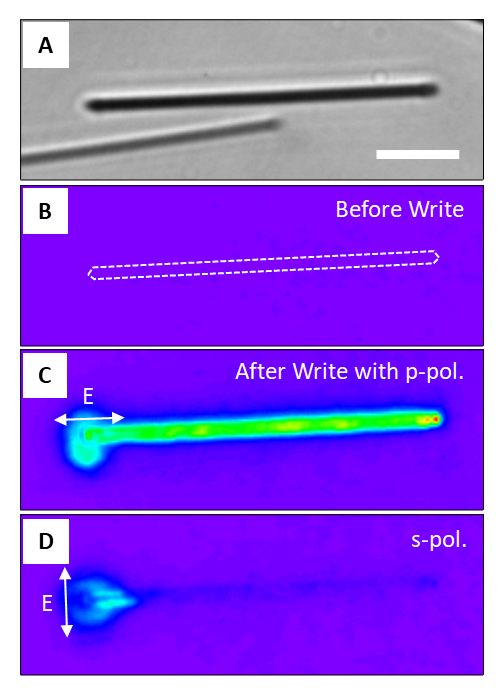


**Figure S8. Write laser polarization dependence of DAE fluorescence recovery on AgNWs.** (A) A transmission image of a DAE/AgNW used in this experiment. The scale bar is 2.5 μm. (B-D) Fluorescence image before (B) and after Write laser irradiation with p-polarization (C) and s-polarization (D).

**§9. Photostationary state kinetics**

Mori et al. have reported similar one-color reversible multiphoton photochromic reactions in an amorphous film of a DAE derivative,^[8]^ deriving expressions for the ratio of the number of open and closed form molecules present, $N_{O}$ and $N_{C}$, respectively. $N_{O}$ and $N_{C}$ at the photostationary state (PSS) can be expressed as:

$-\frac{\partial N_{O}}{\partial t}= N_{O}I^{n}\delta^{\left( n \right)}\Phi_{O\to C}-N_{C}I^{m}\delta^{\left( m \right)}\Phi_{C\to O}=0$ (1)

$-\frac{\partial N_{C}}{\partial t}= N_{C}I^{m}\delta^{\left( m \right)}\Phi_{C\to O}-N_{O}I^{n}\delta^{\left( n \right)}\Phi_{O\to C}=0$ (2)

Here, *I* is the intensity of the laser pulse. $\delta^{\left( n \right)}$ and $\delta^{\left( m \right)}$ are the n- and m-photon absorption cross sections for the open and closed form, respectively. $\Phi_{O\to C}$ and $\Phi_{C\to O}$ are the cyclization and cycloreversion quantum yields. From equation (1) and (2) and the relation $N_{O}+N_{C}=N_{total}$, equation (3) can be obtained:

$\frac{N_{C}}{N_{O}}=\frac{N_{C}}{N_{total}-N_{C}}=\frac{\Phi_{O\to C}}{\Phi_{C\to O}}\times\frac{\delta^{\left( n \right)}}{\delta^{\left( m \right)}}\times I^{(n-m)}$ (3)

By considering that *n* and *m* are 3 and 2, for 3-photon cyclization and 2-photon cycloreversion reactions respectively, the relation between $N_{C}$/$N_{O}$ and *I* is predicted to be linear:

$\frac{N_{C}}{N_{O}}\propto I$ (4)

The measured fluorescence intensity $F(I)$ is directly related to the concentration of the closed form of DAE only. From equation (3), $F(I)$ can be expressed as:

$F(I)\propto N_{C}=(N_{total}-N_{C})\times\frac{\Phi_{O\to C}}{\Phi_{C\to O}}\times\frac{\delta^{\left( n \right)}}{\delta^{\left( m \right)}}\times I^{(n-m)}$ (5)

Assuming that $N_{total}$ is extremely large compared to $N_{C}$, $N_{total}-N_{C}\approx N_{total}$, and $F(I)$ is also predicted to have a linear relation to *I*.

$F(I)\propto N_{total}\times\frac{\Phi_{O\to C}}{\Phi_{C\to O}}\times\frac{\delta^{\left( n \right)}}{\delta^{\left( m \right)}}\times I^{(n-m)}$ (6)

The latter assumption is supported by the following experimental observation: After irradiation of the entire sample, including both the AgNWs and the surrounding regions, with 365 nm UV light for 30 minutes, conditions under which almost all DAE molecules are expected to convert from the non-fluorescent open form to the fluorescent closed form, we observed strong fluorescence. In contrast, after converting most DAE molecules to the non-fluorescent open form by irradiating the entire sample with visible light (488 nm, 3 mW for over 10 minutes), subsequent Write laser irradiation (820 nm, 120 fs, p-polarized, 28.9 GW/cm² for 1 s), which selectively converted DAE molecules around the AgNW to the fluorescent closed form, resulted in a fluorescence intensity that was reduced to less than 1/20 of that observed after the 365 nm UV irradiation. This substantial difference indicates that the Write laser irradiation switches only a small fraction—less than 5%—of the DAE molecules to the fluorescence closed form. Therefore, we consider the assumption to be valid.

**§10. Stability of the AgNW optical memory during Readout laser application**

Here we demonstrate that the Readout laser irradiation (1164 nm, 200 fs, p-polarized, 63.8 kW/cm^2^) barely induces any multiphoton photochromism, causing a negligible change in the PSS of DAE thanks to its weaker power and longer wavelength compared to the Write and Erase lasers. **Figure S9**A shows a transmission image of a DAE/AgNW used in this demonstration. The DAE in the sample was switched to non-fluorescent open form in advance of the Readout laser irradiation (see Section 1). The fluorescence image of the DAE/AgNW exhibits no fluorescence (Figure S9B). The Readout laser was focused on the left end of the DAE/AgNW, which caused fluorescence recovery only at the left end (Figure S9C). Fluorescence recovery along the AgNW body remained at negligible levels. After 10 seconds of Readout laser irradiation, a small degree of DAE cyclization is observed in the vicinity of the excitation site. This localized photoresponse is attributed not to near-field effects but to the far-field component of the Readout laser, which is scattered from the apex of the AgNW and extends beyond the immediate focal region. Although the far-field intensity is significantly weaker than near-field SPP enhancements, it can still initiate detectable cyclization in the surrounding area under prolonged exposure. Next, the DAE along the whole AgNW was switched to the fluorescent closed form by irradiating with the Write laser (820 nm, 120 fs, 80 MHz, p-polarized, 23.9 GW/cm^2^, 1 s) in advance of the Readout laser irradiation. A clear fluorescence image of the DAE/AgNW was obtained (Figure S9D). The Readout laser was again irradiated at the left end of the DAE/AgNW, causing almost no change in fluorescence intensity (Figure S9E).

**Figure S10** shows the time course of SHG enhancement, including the Write and Erase steps (red and blue highlighted, respectively). The SHG enhancement was unchanged during the Readout laser irradiation between the Write/Erase steps, thanks to the thermal stability of DAE molecules and the weak power and long wavelength of the Readout laser. Thus, it is confirmed that the Readout laser does not cause any considerable change in the PSS of DAE, and the SPP propagation efficiency along the AgNW.

Note that irradiation with the 820 nm laser used for the Write and Erase steps can also induce plasmonic SHG at the distal end and can be used for Readout. That is Write, Erase, and Readout steps can all be achieved by a single-color laser, However, the 820 nm laser easily induces multiphoton photochromic reactions because the wavelengths corresponding to two-photon (~ 410 nm) and three-photon (~ 273 nm) absorption of this 820 nm laser are in the absorption spectral range for the open and closed forms of the DAE, respectively. Therefore, lower Readout laser power would be required to avoid unwanted multiphoton photochromic reactions during Readout, making it difficult to observe the plasmonic SHG of 820 nm at the distal end with a high signal-to-noise ratio. Using a separate 1164 nm laser for Readout is thus preferable as not only does its two- and three-photon excitations not overlap with the DAE open and closed forms, its SPP propagation efficiency is greater than that of 820 nm light. Thus, much higher intensity 1164 nm Readout laser can be applied, resulting in higher signal-to-noise distal SHG observed.


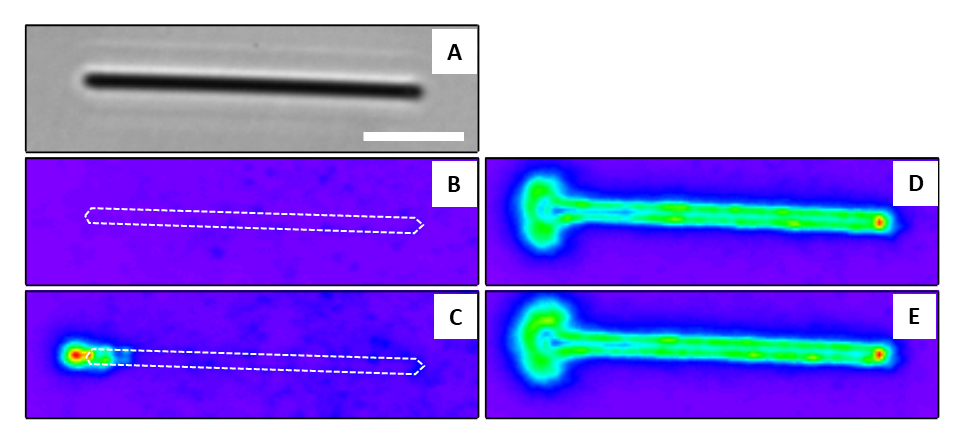


**Figure S9. Stability of the DAE/AgNW PSS during Readout laser irradiation.** (A) A transmission image of a DAE/AgNW was used in this experiment. The scale bar is 2.5 μm. (B and C) Fluorescence images of the AgNW covered by non-fluorescent open form of the DAE (no Write laser irradiation) obtained before (B) and after (C) Readout laser irradiation focused at the left end of the wire (1164 nm, 200 fs, p-polarized, 63.8 kW/cm^2^ for 10 s irradiation). (D and E) Fluorescence images of the AgNW covered by fluorescent closed form of the DAE (following Write laser irradiation, 820 nm, 120 fs, p-polarized, 179 MW/cm^2^ for 1 s) obtained before (D) and after (E) the same Readout laser irradiation.


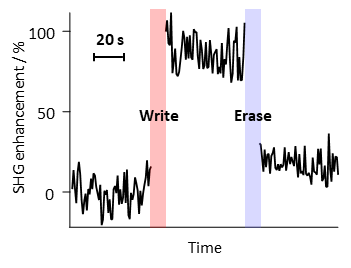


**Figure S10. Time course measurements of the SHG enhancement during Readout laser irradiation.** Plasmonic SHG singles were measured for each 1 s, and the time course of SHG enhancement is plotted including a period of Write laser application (23.9 GW/cm^2^ for 1 s, red highlight) and Erase laser application (2.69 GW/cm^2^ for 1 min, blue highlight).

**§11. AgNW length dependence of DAE/AgNW SHG enhancement**

The propagating SPPs along AgNWs decay exponentially due to the Ohmic dumping.^[1,2]^ **Figure S11**A shows SPP decays assuming decay length, τ ~ 22.2 (± 8.4) μm (black line). The decay length was determined by fluorescence imaging as described in Section 2. Upon Write/Erase laser irradiation, the PSS of DAE is shifted, resulting in a change in the SPP propagating efficiency. In other words, the decay length is modified. Figure S11A also shows SPP decays assuming 20%, 50%, 100%, and 300% increase of decay length as blue, green, orange and red lines, respectively, as could be induced by the Write laser application. Based on the SPP decays, SHG enhancements with those decay length increases are calculated and plotted as a function of AgNW length in Figure S11B. The predicted SHG enhancements show an exponential increase with increase of AgNW length. With 300% increase of decay length, SHG enhancement reaches more than 200% at an AgNW length of 15 μm. To examine the AgNW length dependence of the SHG enhancement experimentally, we checked several DAE/AgNWs, having various wire lengths ranging 3.2 to 18.8 μm. Figure S11C to F show some transmission images of the DAE/AgNWs of different lengths used in these experiments. Figure S11G plots the obtained SHG enhancement induced by Write laser irradiation (820 nm, 120 fs, p-polarized, 179 MW/cm^2^ for 1 s) as a function of AgNW length. The experimentally obtained SHG enhancement reaches almost 200% for an AgNW of length 15 μm, consistent with the predicted 300% increase. However, the SHG enhancement increases linearly with wire length, while the predicted SHG enhancement increases exponentially. This discrepancy between our prediction and experiment could be due to (1) the PSS of DAE being inhomogeneous along the AgNW due to decay of SPPs excited by the Write laser irradiation, and/or (2) the excitation efficiency for plasmonic SHG with the remote excitation configuration (See Section 4) being reduced by increasing distance between the excitation and detection points.


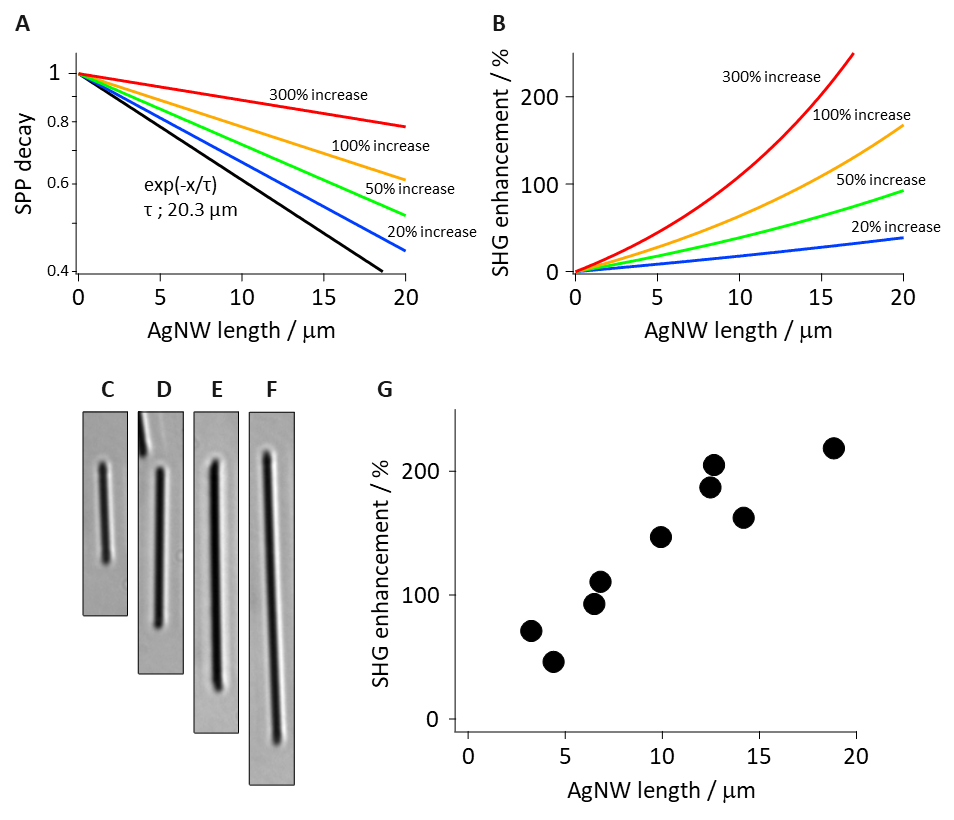


**Figure S11. AgNW length dependence of DAE/AgNW distal-end SHG enhancement.** (A) Exponential decay of propagating SPPs along a AgNW excited by 1164 nm laser at one end, assuming a 1/e decay length τ ~ 20.3 μm (black line). Blue, green, orange and red lines represent SPP decays assuming 20%, 50%, 100% and 300% increase of decay length, respectively, as occurs due to Write laser irradiation and modification of the DAE PSS. (B) Predicted SHG enhancement based on the SPP decay length increase shown in A. (C-F) Transmission images of AgNWs used in this experiment, having length of 4.4 μm (C), 6.8 μm (D), 9.9 μm (E) and 12.5 μm (F). (G) SHG enhancement induced by a Write laser irradiation (820 nm, 120 fs, p-polarized, 179 MW/cm^2^ for 1 s) as a function of AgNW length.

**§12. Polarization-controlled selective fluorescence recovery on closely spaced AgNWs**

**
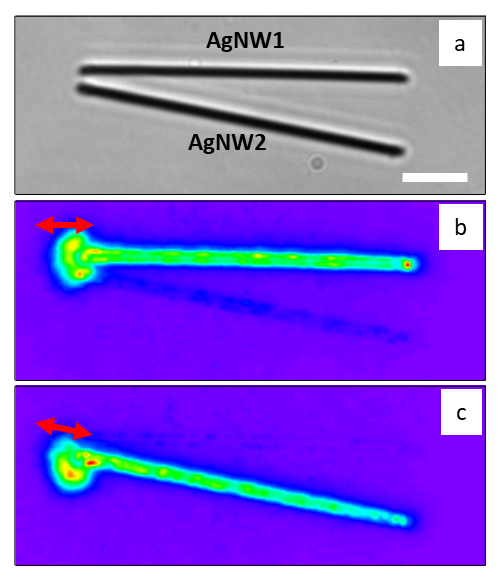
**

**Figure S12. Polarization-controlled selective fluorescence recovery on closely spaced AgNWs.** (a) Transmission image of two DAE-coated AgNWs (AgNW1 and AgNW2) with closely positioned apexes. The scale bar represents 2.5 μm. (b, c) Fluorescence images after Write laser irradiation (the polarization direction is indicated by red arrows). Selective fluorescence recovery was induced only on AgNW1 in (b) and only on AgNW2 in (c), by precisely controlling the alignment and polarization of the Write laser. Despite the apexes of AgNW1 and AgNW2 being in close proximity (~681 nm), which is close to the diffraction limit of the optical system (~671 nm), individual addressability was achieved.

REFERENCES

1. B. Wild, L. Cao, Y. Sun, B. P. Khanal, E. R. Zubarev, S. K. Gray, N. F. Scherer, M. Pelton, *ACS Nano* **2012**, *6*, 472–482.

2. S. Zhang, H. Xu, *ACS Nano* **2012**, *6*, 8128–8135.

3. J. P. Hermann, J. Ducuing, *Opt. Commun.* **1972**, *6*, 101–105.

4. M. Irie, T. Fukaminato, K. Matsuda, S. Kobatake, *Chem. Rev.* **2014**, *114*, 12174–12277.

5. K. Uno, H. Niikura, M. Morimoto, Y. Ishibashi, H. Miyasaka, M. Irie, *J. Am. Chem. Soc.* **2011**, *133*, 13558–13564.

6. H. Sotome, T. Nagasaka, K. Une, C. Okui, Y. Ishibashi, K. Kamada, S. Kobatake, M. Irie, H. Miyasaka, *J. Phys. Chem. Lett.* **2017**, *8*, 3272–3276.

7. P. Walke, S. Toyouchi, M. Wolf, W. Peeters, S. R. Prabhu, T. Inose, S. De Feyter, Y. Fujita, H. Uji-i, *J. Phys. Chem. Lett.* **2018**, *9*, 7117–7122.

8. K. Mori, Y. Ishibashi, H. Matsuda, S. Ito, Y. Nagasawa, H. Nakagawa, K. Uchida, S. Yokojima, S. Nakamura, M. Irie, *J. Am. Chem. Soc.* **2011**, *133*, 2621–2625.
